# Assessment of tau phosphorylation and β‐amyloid pathology in human drug‐resistant epilepsy

**DOI:** 10.1002/epi4.12744

**Published:** 2023-04-24

**Authors:** Alisha Aroor, Phuoc Nguyen, Yibo Li, Rohit Das, Joaquin N. Lugo, Amy L. Brewster

**Affiliations:** ^1^ Department of Psychological Sciences Purdue University West Lafayette Indiana USA; ^2^ Department of Biological Sciences Southern Methodist University Dallas Texas USA; ^3^ Department of Neurology UT Southwestern Medical Center Dallas Texas USA; ^4^ Department of Psychology and Neuroscience Baylor University Waco Texas USA

**Keywords:** Alzheimer's disease, mTOR, refractory epilepsy, tau protein, β‐amyloid

## Abstract

**Objective:**

Epilepsy can be comorbid with cognitive impairments. Recent evidence suggests the possibility that cognitive decline in epilepsy may be associated with mechanisms typical of Alzheimer's disease (AD). Neuropathological hallmarks of AD have been found in brain biopsies surgically resected from patients with drug‐resistant epilepsies. These include hyperphosphorylation of the tau protein (p‐tau) that aggregates into neuropil threads (NT) or neurofibrillary tangles (NFT), as well as the presence of β‐amyloid (Aβ) deposits. While recent studies agree on these AD neuropathological findings in epilepsy, some contrast in their correlation to cognitive decline. Thus, to further address this question we determined the abundance of p‐tau and Aβ proteins along with their association with cognitive function in 12 cases of refractory epilepsy.

**Methods:**

Cortical biopsies surgically extracted from the temporal lobes of patients with refractory epilepsy were processed for immunohistology and enzyme‐linked immunoassays to assess distribution and levels, respectively, of p‐tau (Antibodies: Ser202/Thr205; Thr205; Thr181) and Aβ proteins. In parallel, we measured the activation of mechanistic target of rapamycin (mTOR) via p‐S6 (Antibodies: Ser240/244; Ser235/236). Pearson correlation coefficient analysis determined associations between these proteins and neurophysiological scores for full‐scale intelligence quotient (FSIQ).

**Results:**

We found a robust presence of p‐tau (Ser202/Thr205)‐related NT and NFT pathology, as well as Aβ deposits, and p‐S6 (Ser240/244; Ser235/236) in the epilepsy biopsies. We found no significant correlations between p‐tau (Thr205; Thr181), Aβ, or mTOR markers with FSIQ scores, although some correlation coefficients were modest to strong.

**Significance:**

These findings strongly support the existence of hyperphosphorylated tau protein and Aβ deposits in patients with human refractory epilepsy. However, their relation to cognitive decline is still unclear and requires further investigation.

**Key Points**

- Refractory epilepsy can be comorbid with cognitive dysfunctions.
- Hyperphosphorylated‐tau and β‐amyloid proteins are found in human epilepsy.
- Abundance of p‐tau or β‐amyloid does not correlate with cognitive decline in epilepsy.

## 1 INTRODUCTION

Epilepsy is characterized by the occurrence of two or more spontaneous seizures likely triggered by imbalances of excitatory‐inhibitory signals in the brain.^1^ Extensive evidence supports that genetic and/or acquired factors can promote the development of epilepsy.^2^, ^3^ An increased risk for epilepsy can result from genetic mutations, developmental brain malformations, traumatic brain injuries, cerebral infections, status epilepticus, and stroke, among others.^3^ It is estimated that of the 70 million people diagnosed with epilepsy worldwide 30%‐40% have drug‐resistant seizures,^4^ and 50% experience additional health conditions that can include psychiatric and cognitive impairments.^5^ For example, in people with epilepsy, the prevalence of depression is 20%‐35%^6^, ^7^ and anxiety 10%‐25%.^7^, ^8^ Individuals with medically refractory epilepsy are at higher risk (70%‐80%) for the development of cognitive comorbidities including memory loss and attention difficulties.^9^ Although the underlying causes for the cognitive impairments are not definitively known, recent evidence suggests that overlapping mechanisms with Alzheimer's disease (AD) pathology may play a role.^10^, ^11^, ^12^

Alzheimer's disease is a neurodegenerative disorder that is characterized by a progressive cognitive decline that results in memory loss and dementia.^13^ Histopathological hallmarks of AD include the accumulation of β‐amyloid (Aβ) plaques and the presence of neuropil threads (NT), or neurofibrillary tangles (NFT) formed by the aggregation of hyperphosphorylated tau protein (p‐tau). The accumulation of Aβ plaques and NFT can disrupt neural function and promote neurodegeneration leading to cognitive decline and dementia,^13^ neuronal hyperexcitability^14^, ^15^ and seizures.^11^, ^16^ Contemporary evidence supports that the occurrence of epilepsy is higher in AD patients with severe dementia.^11^ The reported incidence of unprovoked seizures or epileptiform activity in AD patients varies in different studies ranging from a low of 9%‐10%^17^, ^18^ to a high of 16%‐38%.^19^, ^20^, ^21^

Recent evidence supports the presence of AD‐like pathology in human refractory epilepsy.^22^, ^23^, ^24^, ^25^, ^26^ Histopathological studies found evidence of p‐tau and Aβ pathology in brain tissues surgically resected from epilepsy patients with drug‐resistant seizures that include temporal lobe epilepsy (TLE)^22^, ^26^, ^27^ or focal cortical dysplasia (FCD).^24^ TLE is associated with disruptions/injury in the temporal lobes and internal structures including the hippocampus and amygdala.^28^ FCD is associated with malformations of cortical development characterized by abnormal cytoarchitecture of the cortex with reduced neuronal densities and enlarged dysmorphic cells.^29^ In biopsies from TLE cases, two studies reported robust tau‐related NT and NFT accumulation that correlated with cognitive deficits^22^, ^26^ while a more recent study found sparse p‐tau with no correlation to cognitive impairment.^27^ Evidence of Aβ pathology in both TLE and FCD is less clear due to contrasting findings of high^22^, ^24^ and low abundance of Aβ‐related pathology.^27^ Thus, to assess the extent of AD‐like pathology and its relation to cognitive dysfunction in epilepsy, we measured the relative abundance of p‐tau and Aβ proteins in brain biopsies surgically resected from 12 patients diagnosed with refractory epilepsy and correlated these to neurophysiological scores for full‐scale intelligence quotient (FSIQ) for each case. In parallel, we measured activation of the mechanistic target of rapamycin (mTOR) because enhanced signaling of this pathway can occur in response to neuronal hyperactivity and seizures in both human and experimental epilepsy,^30^, ^31^, ^32^, ^33^ and contributes to the pathology of FCD^34^ and AD.^35^

## 2 METHODS

### 2.1 Ethics statement

Brain tissues were collected with patients' informed consent under the Institutional Review Board (IRB) protocol #1011004282 (Development of a Biorepository for Methodist Research Institute; Indiana University Health Biorepository). All identifiable information was removed prior to performing experiments and analyses conducted under the IRB protocol #1507016240 (Purdue University) and the IRB protocol #21‐126 (Southern Methodist University).

### 2.2 Human brain tissues

Surgically resected cortical tissues were derived from patients (P) with a history of medically refractory epilepsy (*n* = 12) (Table 1). All biopsies utilized for this study are from the temporal cortex, no hippocampi included. MRI scans indicated epilepsy involving the temporal lobes, with cases with presumptive mesial temporal sclerosis with cortical dysplasia (P1, P9), presumptive FCD type I (P3), presumptive mesial temporal sclerosis (P5), possible cortical dysplasia (P6), possible neoplasm, FCD, or focal cortical heterotopia (P8), and non‐specific (P7, P10, P11). Imaging and pathology findings indicated FCD type Ib for P2 and FCD type IIIa for P4 and P12.

**TABLE 1:** Neurophysiological and anatomical information from resected brain biopsies from patients with refractory epilepsy.

Patient #	Age at surgery (years)	Sex	Tissue origin	FCD	Epilepsy duration (years)	FSIQ
1	24	Male	Right temporal lobe	NA	6.4	106
2	25	Female	Right temporal lobe	Ic	12.0	NA
3	25	Female	Left lateral temporal lobe	NA	24.2	76
4	28	Male	Left lateral temporal lobe	IIIa	26.4	90
5	36	Female	Lateral temporal lobe	NA	34.3	74
6	43	Female	Right anterior temporal lobe	NA	39.1	87
7	44	Male	Right temporal lobe	NA	34.0	93
8	51	Male	Temporal Lobe	NA	3.5	NA
9	53	Female	Right temporal Lobe	NA	51.8	Average
10	55	Male	Lateral temporal cortex	NA	20.0	114
11	59	Male	Left temporal lobe	NA	31.0	Average/Low
12	67	Male	Right temporal lobe	IIIa	1.1	82

### 2.3 Neurocognitive evaluation

As part of routine pre‐surgical workup, patients underwent neuropsychological evaluation with a qualified neuropsychologist administering the Wechsler Abbreviated Scale of Intelligence Test. FISQ scores were estimated from this test and were available for eight patients; these scores were used in correlational analyses with neuropathological findings. FSIQ scores were not available (NA) for two patients, P2 and P8. Two patients had descriptive reports without FSIQ. The neurocognitive assessment of P9 and P11 described them as average and low average, respectively (Table 1). These were not used in the correlational analyses.

### 2.4 Tissue processing for immunoassays

Brain biopsies were either placed into cryovials, submerged in liquid nitrogen, and stored at −80°C or placed in formalin for fixation after surgical resection. For immunostaining, fresh frozen samples were fixed in ice‐cold 4% paraformaldehyde (PFA) at 4°C overnight. For cryoprotection, all formalin and PFA‐fixed tissues were placed in 30% sucrose diluted in 1X Phosphate Buffer Solution (PBS) at 4°C for 48‐72 hours. Then, tissues were frozen in dry ice and stored at −80°C until used for immunostaining. Brain tissues were sectioned (40 μm) using a Leica CM1860 cryostat and stored in 1XPBS with Sodium Azide (0.01%) at 4°C. For biochemical analyses for immunoassays, cortical brain biopsies containing white and gray matter (~1.5 cm pieces) were homogenized using ice‐cold 1XPBS with a proteinase inhibitor cocktail (P2850, P8340, Sigma‐Aldrich). Protein concentration was determined with Bradford assay (5000205, Bio‐Rad) and all samples were diluted to similar protein concentrations (1 mg/mL). Samples were kept on ice at all times.

### 2.5 Immunohistochemistry

For Immunohistochemistry (IHC) staining, 3‐4 sections per brain were processed following previously described protocols.^36^ Sections were incubated in 1XPBS‐3% Triton (T) overnight at 4°C, followed by 3% Hydrogen Peroxide (30 minutes), a wash in 1XPBS‐3%T at room temperature (RT), and incubation in immuno buffer (5% goat serum, 0.3% BSA, 1XPBS‐3%T) overnight at 4°C. Incubation in primary antibodies was done overnight at 4°C [Aβ (rabbit, 1:100; ab2539; Abcam), p‐tau (Ser202, Thr205) (AT8) (mouse, 1:100; MN1020; Invitrogen), p‐S6 (Ser240/244) (rabbit, 1:100; #5364; Cell Signaling), and NeuN (rabbit, 1:100; Abcam)]. Tissues were then washed in 1XPBS‐0.1%T (3 × 10 minutes), incubated in biotinylated secondary antibodies (anti‐rabbit, 1:500; BA‐1000, or anti‐mouse, 1:500; BA‐9200; Vector Laboratories) (1 hour), washed in 1XPBS‐0.1%T (3 × 10 minutes), and developed using the Avidin/Biotin Complex solution (30 minutes) and DAB staining (Vector Laboratories) following manufacturer's instructions. Brain sections were mounted on gelatin‐coated glass slides, Nissl stained, dehydrated in ethanol solutions [50%, 70%, 95%, 100%], de‐fatted in Xylene, and coverslipped using Permount mounting medium. The immunostaining signal was visualized using a Leica DM500 microscope equipped with a high‐resolution digital camera (Leica MC120 HD), or a Nikon Ti2‐E equipped with a high‐resolution digital camera (DS‐Ri2 Color CMOS camera). Immunostaining for the different proteins of interest was similar in both formalin and PFA‐fixed tissues.

### 2.6 Enzyme‐linked immunoassay (ELISA)

Protein levels of Aβ1‐40 were measured using a human Amyloid beta 1‐40 ELISA Kit (Abcam human Amyloid beta 1‐40 ELISA Kit, #AB193692) following manufacturer's instructions. Equal protein concentrations, standard or blank were added to each well of the 96‐well plate followed by incubation for 2.5 hours at RT. Following four washes, samples were incubated with biotinylated Human Amyloid beta 1‐40 detection antibody for 1 hour at RT. Following 4 washes, samples were incubated in HRP‐Streptavidin solution for 45 minutes at RT and washed again (4×). TMB substrate solution was then added, and samples were intubated in the dark for 30 minutes at RT. The reaction was stopped and read immediately at 450 nm using the CLARIOstar software. Results are shown as ng/mL.

Relative protein levels of p‐tau (Thr181) and p‐tau (Thr205) were measured using the human p‐tau ELISA Kits [Phospho‐Tau (Thr181) ELISA kit, #58537; Phospho‐Tau (Thr205) ELISA kit, #51580] following manufacturer's instructions. Equal protein concentrations derived from each case, positive control or blank were added to each well of 96‐well plates, then the antibody cocktail was added and incubated on a plate shaker (400 rpm) for 1 hour at RT. Following three washes, TMB substrate solution was added to each well, and samples were incubated in the dark on a plate shaker (400 rpm) for 15 minutes at RT. The reaction was stopped and read immediately at 450 nm using the CLARIOstar software. Results are shown as relative signal intensity (rsi).

### 2.7 Western blotting

All samples were diluted to 1 mg/mL using 4× Laemmli Sample Buffer for SDS‐PAGE with Tris‐glycine gels (12%). Samples were transferred to polyvinylidene fluoride membranes (GE Healthcare), and blocked for 1 hour with 5% non‐fat milk diluted in 1XPBS on a rocking platform at RT. Incubation with primary antibodies was done overnight at 4°C: p‐S6 (Ser240/244) (rabbit, 1:1K; #2215; Cell Signaling), p‐S6 (Ser235/236) (rabbit, 1:1K; #4858; Cell Signaling), S6 (mouse, 1:1K; #2317; Cell Signaling), and Actin (rabbit, 1:1K; A2066; Millipore Sigma). Membranes were washed in 1XPBS‐0.1%Tween and incubated with HRP‐linked secondary antibodies (anti‐rabbit or anti‐mouse, 1:1K; #7074, #7076; Cell Signaling). Immunoreactive bands were visualized using enhanced chemiluminescence prime western blotting detection reagent (GE Healthcare), captured on Double Emulsion Blue Autoradiography Film (Midwest Scientific). Membranes were stripped from primary antibodies using stripping buffer (25 mM glycine, pH 2.0, 10% SDS) and re‐blotted as described above. Note that there was not sufficient protein from P5 for immunoblotting. Image J software (NIH) was used to measure the relative signal intensity of immunoreactive bands for phosphorylated and total proteins for each sample/lane.^37^ Actin was used as a loading control.

### 2.8 Pearson correlation coefficient analyses

GraphPad Prism 9 was used for Pearson correlation coefficient (*r*) analysis. The strength/degree of each association is based on r as follows: Positive: high/large, between 0.5 and 1.0; moderate/medium, between 0.30 and 0.49; low/small between 0.1 and 0.3. Negative: high/large, between −0.5 and − 1.0; moderate/medium, between −0.30 and − 0.49; low/small between −0.1 and −0.3. No correlation, 0. Statistical significance was set at *P <* 0.05. Figures were created using Photoshop.

## 3 RESULTS

**FIGURE 1:**
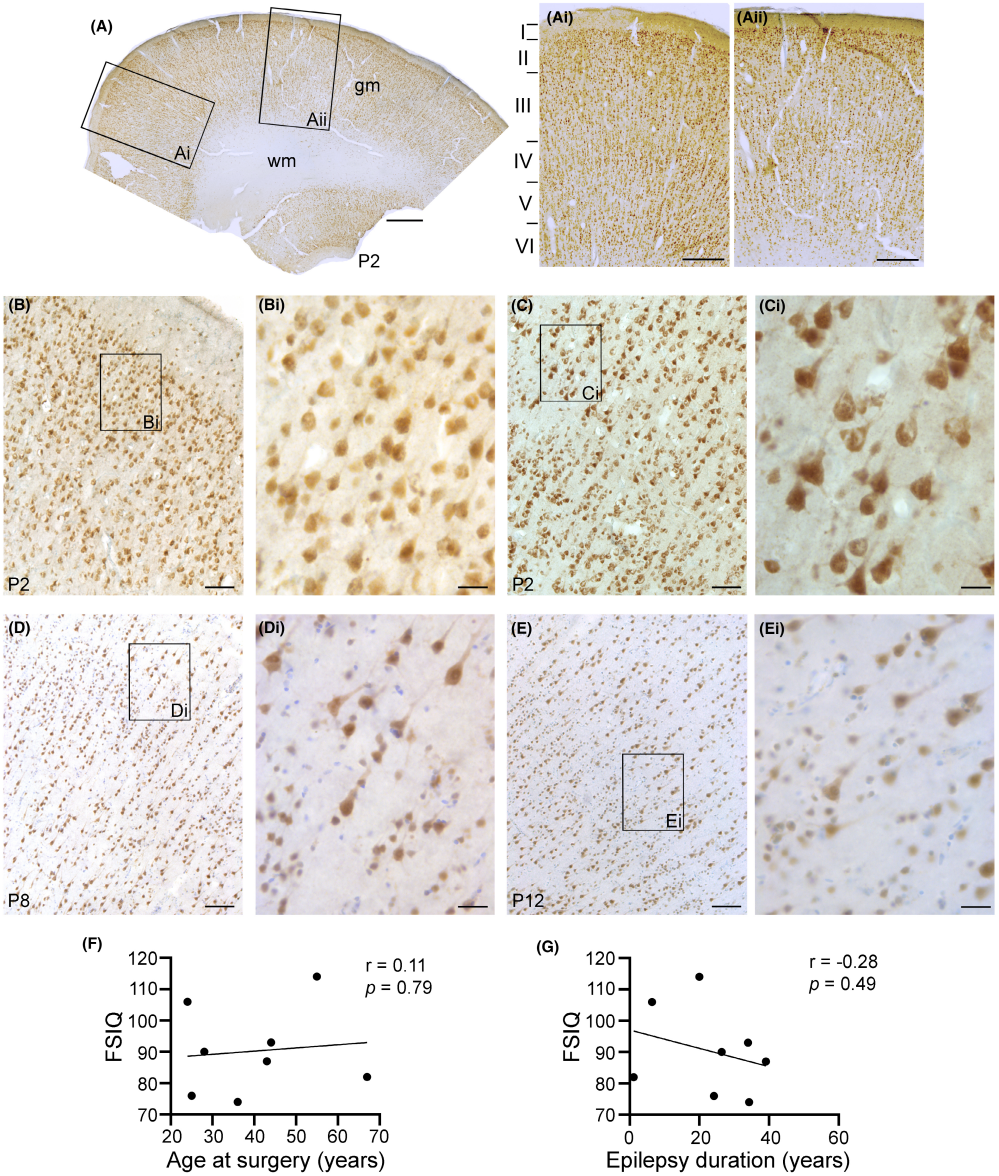
NeuN immunostaining shows altered neuronal densities and abnormal neurons in cortical samples from human epilepsy cases. Representative images immunostained with antibodies against NeuN, a neuronal marker, are shown for cortical tissues surgically resected from different patients (P) with FCD with drug‐resistant seizures (A‐Ei). NeuN immunoreactivity is shown in brown and nuclear Nissl staining is shown in blue. NeuN signal (Ai) shows normal appearing cortical layering along with areas of dispersed NeuN staining (Aii). NeuN staining within the cortex revealed organized cortical layering with normal appearing neurons compared (B‐Bi) to enlarged dysmorphic neurons (C‐Ci) observed within the same patient sample (Patient 2; P2). Representative cortical tissue samples from two additional different patients show NeuN‐positive neuronal staining with characteristics of radial microcolumnar disorganization with abnormal cell size in P8 (D‐Di) and P12 (E‐Ei). Representative higher magnification images containing cortical layers II & III are shown in panels (Bi, Ci, Di, Ei). Scale bars: A, 1 mm; Ai and Aii, 500 μm; (B‐E), 250 μm, and (Bi‐Ei), 25 μm; white matter; wm; gray matter, gm. Pearson correlation coefficient (r) analyses between FSIQ scores and the age at surgery (F) and the epilepsy duration (G) are shown.

In this study, we used human brain tissues derived from 12 different patients with drug‐resistant epilepsy (Table 1). These tissues were obtained from either the right or left temporal lobes of 7 male and 5 female patients (P) whose ages ranged from 24 to 67 years, with a mean age of 42.5 years. To identify the cortical organization of the brain biopsies, we performed immunostaining with antibodies against NeuN, a neuronal marker (Figure 1A). Robust NeuN signal was localized within the gray matter (gm) of the temporal cortices of all samples used in this study. These tissues displayed areas of normal‐appearing cortical organization with distinctive layering I‐VI (Figure 1Ai). Adjacent to the “normal” areas some tissues showed dispersed and disorganized neuronal arrangements (Figure 1Aii) with lower neuronal densities, or abnormal cell size (Figure 1B‐D). Figure 1B shows representative images of normal‐appearing cortical regions and normal‐appearing NeuN‐positive neurons (Figure 1Bi). These were found adjacent to areas of cortical dyslamination (Figure 1C) with enlarged neurons (Figure 1Ci). Other cases also showed a reduced density of NeuN positive neurons (Figure 1D,E) along with radial microcolumnar organizations (Figure 1Di,Ei; FCD IIIa). NeuN staining showed variable neuronal densities along with evidence of radial microcolumnar organization in the different biopsies (data not shown). In this group of cases, we found low correlation coefficients between FSIQ scores and the age at surgery (Figure 1F) or the epilepsy duration (Figure 1G), that were not statistically significant.

**FIGURE 2:**
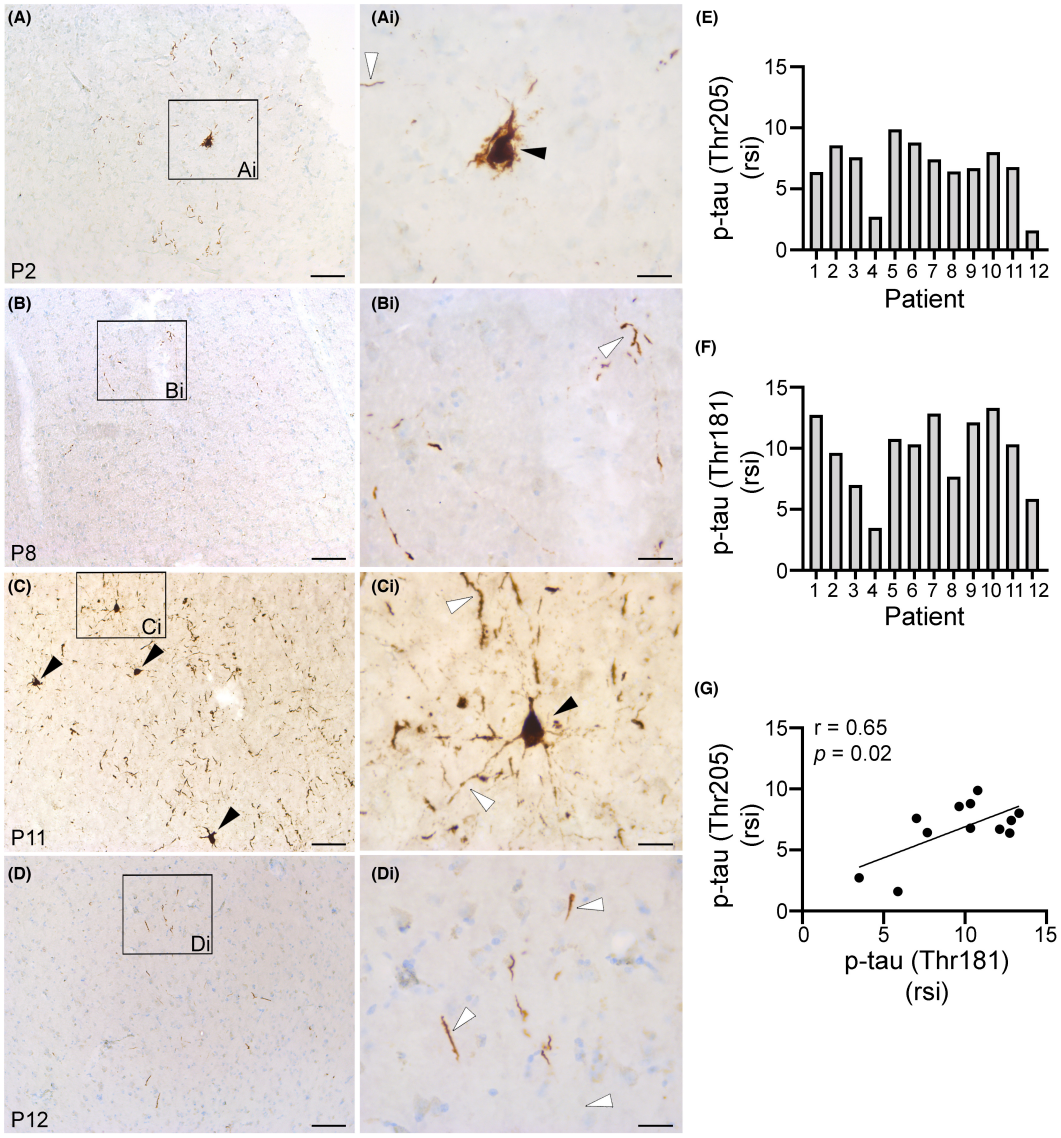
Phosphorylated tau (p‐tau) is abundant in cortical samples from human refractory epilepsy. Representative images immunostained with antibodies against AT8, a marker for p‐tau (Ser202/Thr205), are shown for cortical tissues surgically resected from different patients (P) with drug‐resistant seizures (A‐Di). P‐tau staining is shown in brown while nuclear Nissl staining is shown in blue. P‐tau signal is observed in the form of neurofibrillary tangles localized in pyramidal neuron‐like cells (black arrowheads) and neuropil threads (white arrowheads) within the brain parenchyma (Ai, Bi, Ci, Di). Scale bars: A, B, C, and D, 250 μm; Ai, Bi, Ci, and Di, 25 μm. Protein levels for p‐tau (Thr205) (E) and p‐tau (Thr181) (F) in each brain biopsy are shown as relative signal intensity (rsi). Pearson correlation coefficient (*r*) analysis between both p‐tau phosphorylated sites is shown in (G).

**FIGURE 3:**
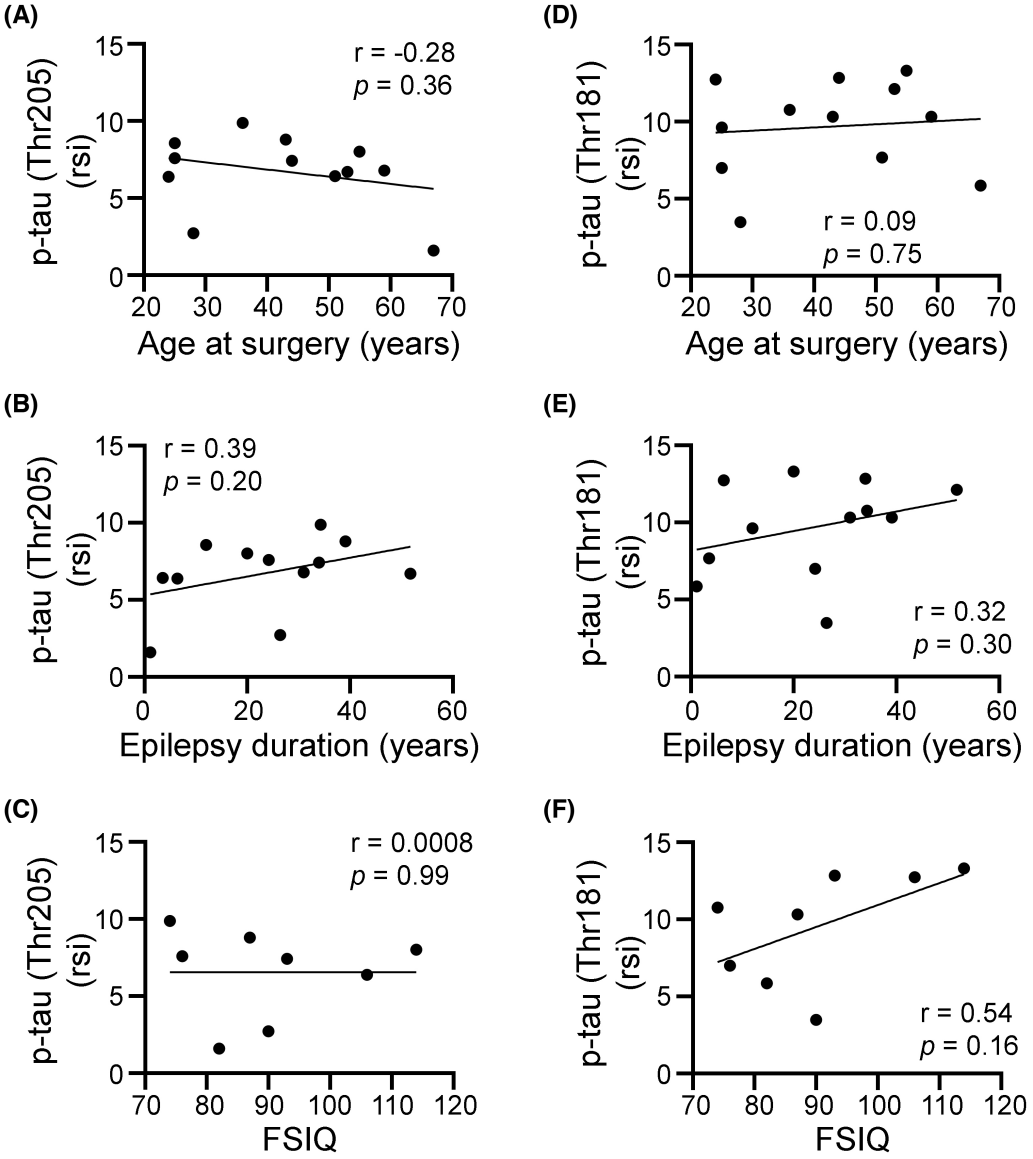
Correlation analysis of p‐tau abundance with age, epilepsy duration, and FSIQ scores. Pearson correlation coefficient (*r*) analysis of p‐tau (Thr205) and p‐tau (Thr181) to age at surgery (A, D), epilepsy duration (B, E), and FSIQ (C, F) are shown.

**FIGURE 4:**
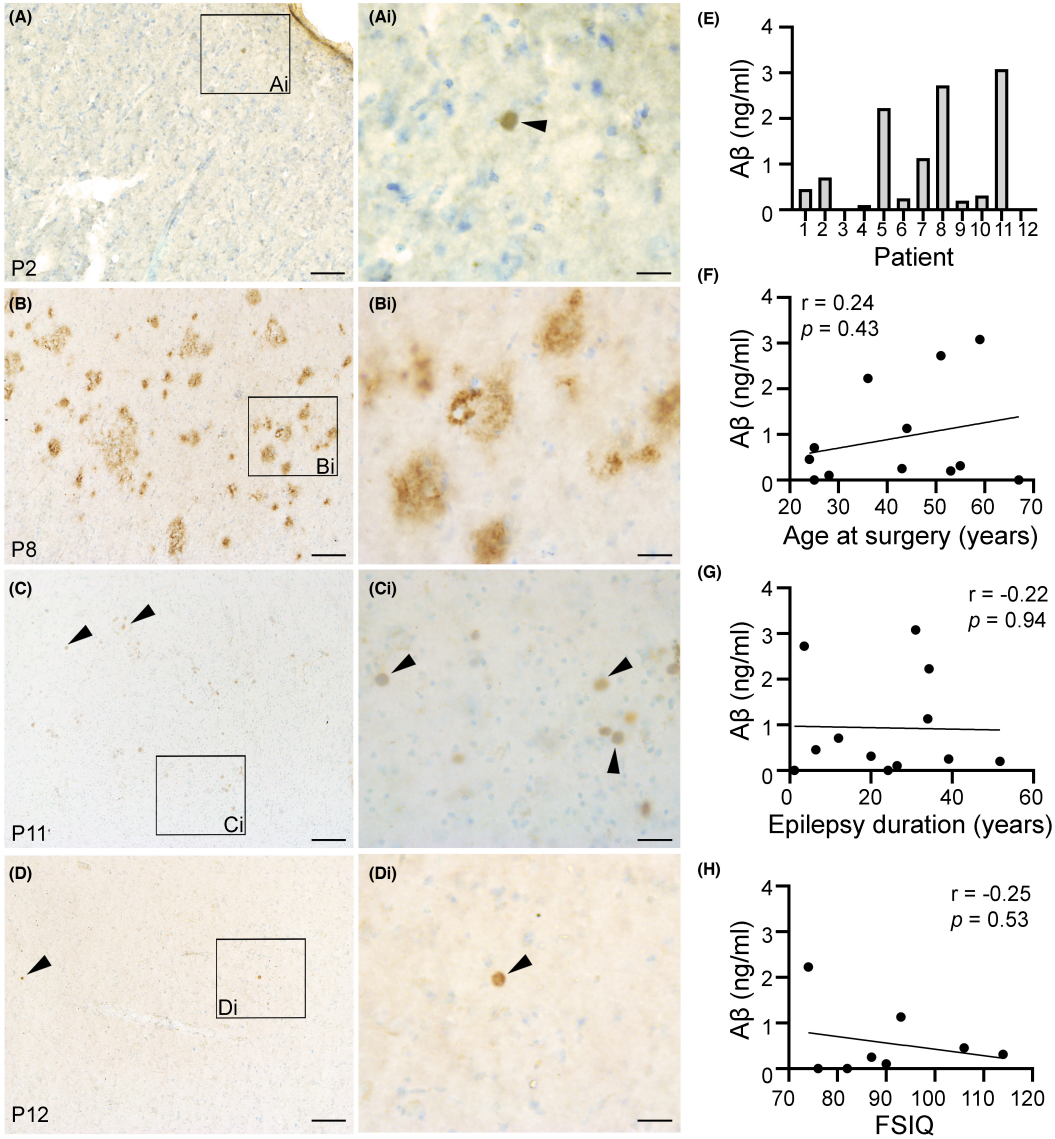
β‐amyloid protein is evident in cortical samples from human refractory epilepsy. Representative images immunostained with antibodies against Aβ are shown for cortical tissues surgically resected from different patients (P) drug‐resistant seizures (A‐Di). Aβ immunoreactivity is shown in brown and nuclear Nissl staining is shown in blue. Variable patterns of Aβ signal are evident in different patient biopsies (P) (A‐Di). Most tissues showed small circular structures positive for Aβ signal (black arrowheads) (A, C, D). One sample displayed a robust accumulation of Aβ in the form of plaques throughout the cortex of the entire sample (B). Representative higher magnification images are shown in panels Ai, Bi, Ci, Di. Scale bars: A, B, C, and D, 250 μm; Ai, Bi, Ci, and Di, 25 μm. Protein levels for Aβ are shown as ng/ml (E). Pearson correlation coefficient (*r*) analysis between Aβ and the age at surgery (F), the epilepsy duration (G), and FSIQ scores (H) are shown.

Next, we investigated the extent of tau phosphorylation (Figures 2 and 3) and Aβ (Figure 4) pathology in epilepsy brain biopsies (Table 1). To determine the spatial distribution of p‐tau we used antibodies against AT8 [p‐tau (Ser202/Thr205)]. Hyperphosphorylated tau protein aggregates to form NT (Figure 2, white arrowheads) or NFT (Figure 2, black arrowheads). We found evidence of both types of p‐tau structures NT and NFT co‐occurring in 6 of the 12 brain biopsies (Figure 2A‐D). To measure the extent of tau phosphorylation we used ELISA tests with antibodies against two tau phosphorylation sites (Thr205 and Thr181) identified in AD patients.^38^ We found variable levels of p‐tau across the 12 biopsies (Figure 2E,F). Consistent with the immunostaining (Figure 2A‐D), a higher abundance of p‐tau was evident in P2 (Figure 2A) and P11 (Figure 2C) relative to P8 (Figure 2B) and P12 (Figure 2D). We found a strong correlation between tau phosphorylation at both sites (Thr205 and Thr181) that was significant (*P* = 0.02) (Figure 2G). Figure 3 shows the correlation between the p‐tau phosphorylated residues (Thr205 and Thr181) and the age at surgery (Figure 3A,D), the epilepsy duration (Figure 3B,E), and the FSIQ scores (Figure 3C,D). The correlation coefficient indicated low to no correlation between p‐tau and the age at surgery (Figure 3A,D). There was a trend toward a moderate to strong association between p‐tau (both sites) and the epilepsy duration (Figure 3B,E) and the FSIQ scores (Figure 3C,F) (*P* > 0.05).

Similar to p‐tau, we found a variable abundance of Aβ protein across the different epilepsy brain biopsies (Figure 4). Eight of the 12 samples showed immunoreactivity for Aβ (Figure 4A‐D). Scarce Aβ immunoreactivity (Figure 4A,D, black arrowheads) was seen in seven samples while only one biopsy showed robust accumulation of Aβ throughout the entire specimen (Figure 4B). We quantified Aβ protein levels in the brain tissue homogenates using ELISA (Figure 4E) and found that the abundance of Aβ protein in each sample was consistent with the histological observations (Figure 4A‐D). For example, a higher abundance of Aβ protein was evident in P8 (Figure 4B) and P11 (Figure 4C) relative to P2 (Figure 4A) and P12 (Figure 4D). Pearson correlation coefficient analyses showed a low correlation between Aβ and the age at surgery (Figure 4F), the epilepsy duration (Figure 4G), or the FSIQ scores (Figure 4H).

**FIGURE 5:**
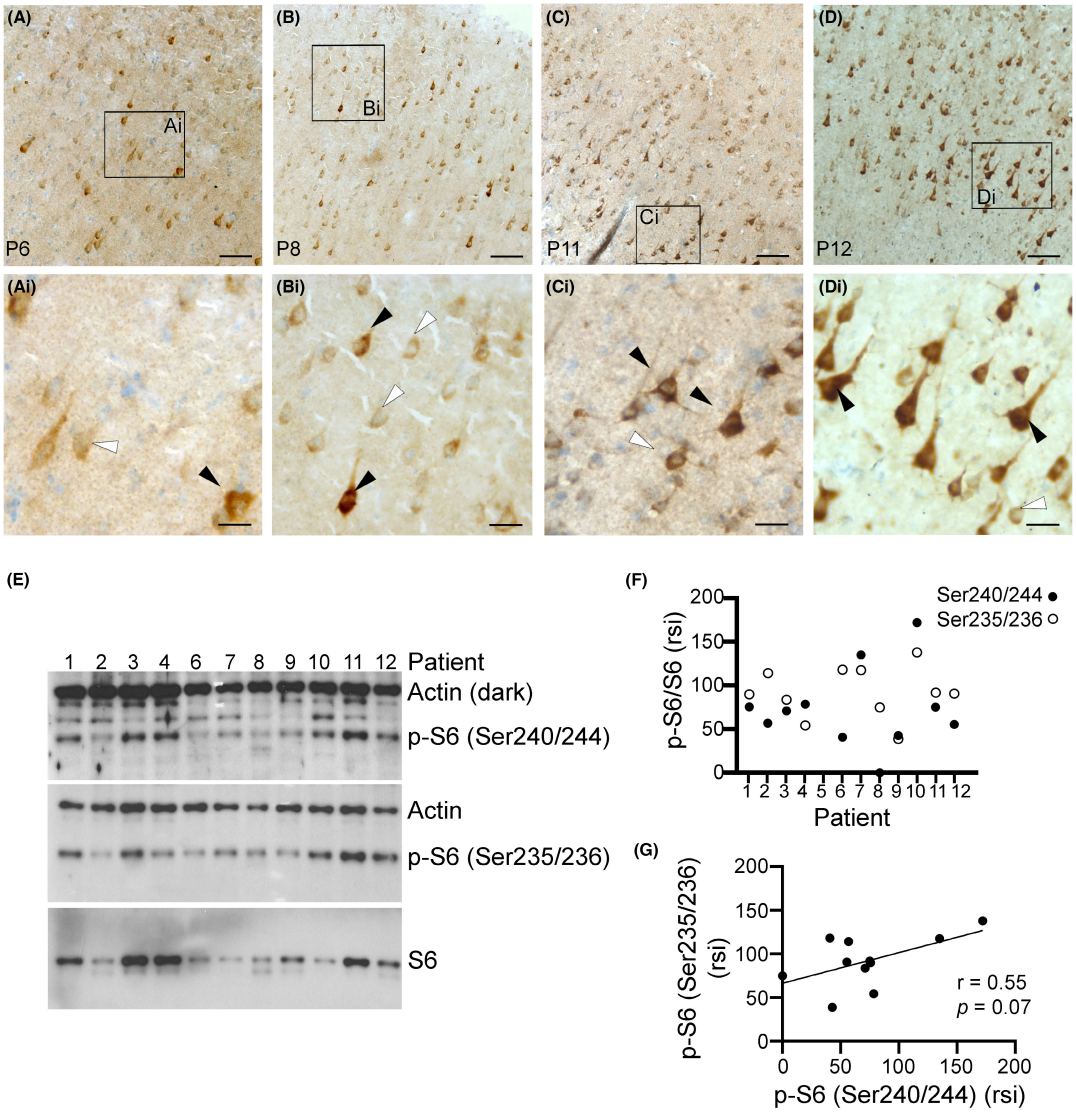
Phosphorylated ribosomal S6 (P‐S6) is evident in cortical samples from human refractory epilepsy. Representative images immunostained with antibodies against p‐S6 (Ser240/244), a marker for mTOR activity, are shown for cortical tissues surgically resected from different patients (P) with drug‐resistant seizures (A‐Di). P‐S6 (Ser240/244) immunoreactivity is shown in brown and nuclear Nissl staining is shown in blue. Variable intensities of p‐S6 signal are evident across the different patient samples (A‐Di). Black arrows point to neurons with robust p‐S6 (Ser240/244) staining while the white arrows point to cells with comparable weaker signal (Ai‐Di). Representative higher magnification images of cortical layer III are shown in panels Ai‐Ci. Scale bars: A, B, C, D, 250 μm; Ai, Bi, Ci, and Di, 25 μm. Representative immunoblots are shown for p‐S6 (Ser240/244), p‐S6 (Ser 235/235), S6, and Actin (loading control) (E). Quantification of the relative signal intensity (rsi) for p‐S6/S6 is shown in (F). Pearson correlation coefficient (*r*) analysis between both p‐S6 phosphorylated sites is shown in (G).

**FIGURE 6:**
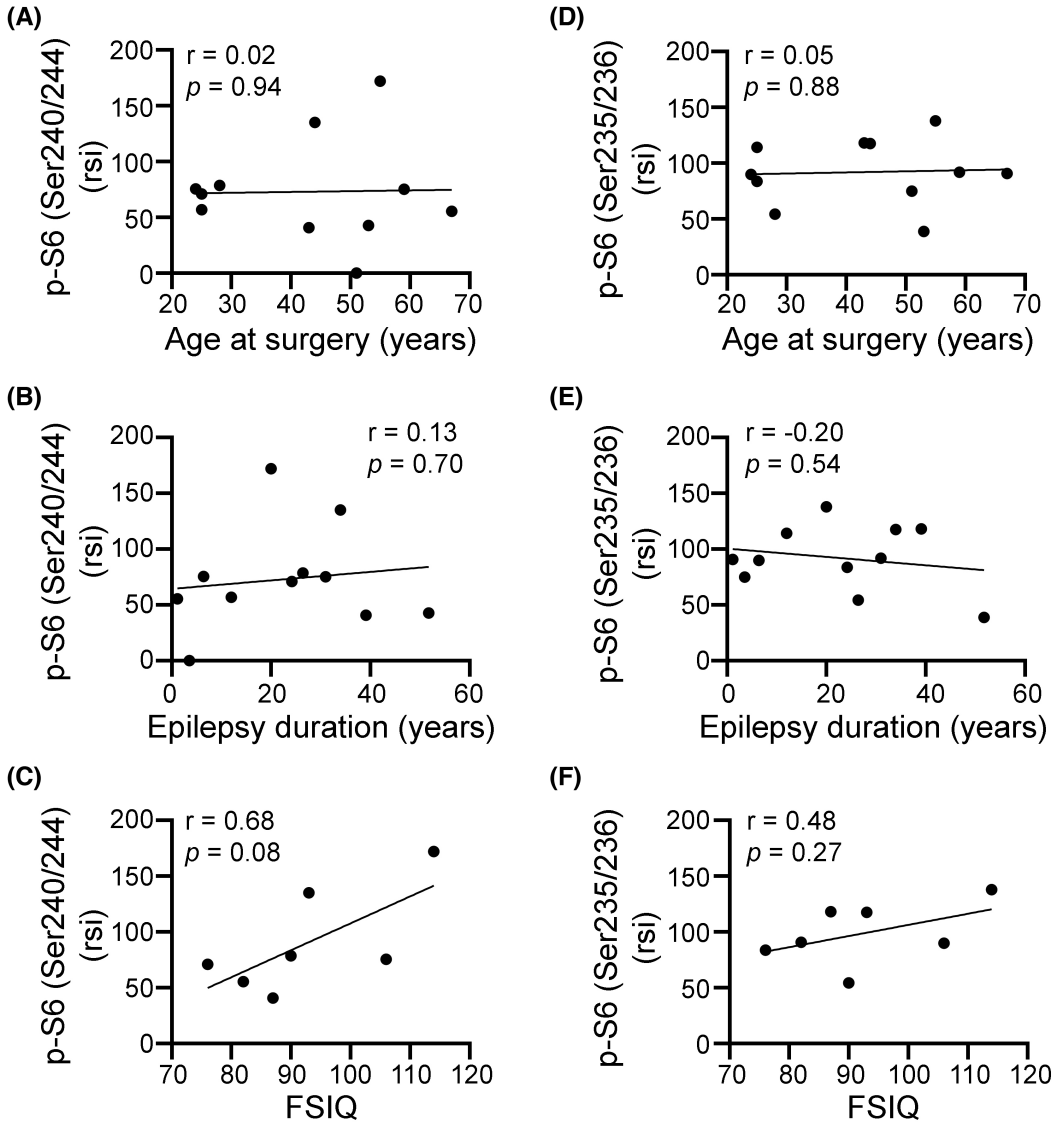
Correlation analysis of p‐S6 abundance with age, epilepsy duration, and FSIQ scores. Pearson correlation coefficient (*r*) analysis of p‐S6 (Ser240/244) and p‐S6 (Ser 235/235) to the age at surgery (A, D), the epilepsy duration (B, E), and FSIQ (C, F) are shown.

Because hyperactivation of the mTOR signaling cascade is associated with neuronal hyperactivity, seizures, epilepsy^30^, ^31^, ^32^, ^33^, ^34^, ^39^ and AD^35^ pathology, we also examined the activation of this pathway in the epilepsy brain biopsies. We used antibodies against phosphorylation of the ribosomal S6 protein at the Ser240/244 site, specific for mTOR complex 1 activation (IHC and WB), and p‐S6 (Ser235/235) (WB), which can be activated by other pathways including ERK.^40^ Prominent p‐S6 (Ser240/244) signal was visible in neurons of all cases (Figure 5A‐D). Stronger p‐S6 (Ser240/244) was evident within regions of cortical dysplasia or low apparent neuronal densities. Note that this p‐S6 (Ser240/244) signal was mainly localized to cells with clear neuronal shapes (Figure 5Ai‐Di). Potential glial localization p‐S6 (Ser 240/244) or immunostaining for p‐S6 (S235/235) were not examined in this study. The abundance of both p‐S6 at both phosphorylation sites (Ser240/244; Ser235/236) was measured in brain tissue homogenates from 11 of 12 cases (Figure 5E,F). We found a strong correlation between phosphorylation of S6 at both sites (Ser240/244; Ser235/236) with *p* = 0.07 (Figure 5G). Abundance of p‐S6 in the epilepsy brain biopsies showed a low correlation to the age at surgery (Figure 6A,D) or the epilepsy duration (Figure 6B,E). Interestingly, there was a trend toward a moderate to strong association between the FSIQ scores and p‐S6 [(Ser240/240) (Figure 6C); (Ser235/235) (Figure 6F)] (*P* > 0.05).

In addition, we compared the extent to which p‐tau and Aβ pathology correlated with each other and to mTOR activation (Figure S1). Low correlation coefficient values were found between the levels of p‐tau and Aβ (Figure S1A,E), moderate correlations between mTOR activation and p‐tau protein levels (Figure S1B,C,F,G), and low to no correlations between mTOR and Aβ protein abundance (Figure S1D,H). These data suggest a strong association between mTOR activation and tau pathology, and that the abundance of Aβ in the epilepsy brain biopsies may be independent of age, epilepsy duration, cognitive function, or mTOR activation.

## 4 DISCUSSION

Our findings showed abundant p‐tau (Ser202/Thr205)‐related NT and NFT pathology, with variable levels of p‐tau at two phosphorylation sites (Thr205 and Thr181) (Figures 2 and 3), Aβ deposits (Figure 4) and mTOR activation (Figures 5 and 6) in brain biopsies from drug‐resistant epilepsy cases. We found no statistically significant correlations between FSIQ and p‐tau, Aβ, or mTOR pathology, or between the abundance of these molecules and the age at surgery or the epilepsy duration. However, according to their correlation coefficient values some of these associations showed moderate to strong correlations that could be comprehensively addressed with a larger group of cases in future follow‐up studies.

In human AD, more than 40 phosphorylatable tau residues have been identified and associated with pathological disruptions in microtubule assembly.^41^ From these residues, only a few have been investigated in human epilepsy, p‐tau (Ser 202),^24^, ^26^, ^42^ p‐tau (Ser202/Thr205),^22^, ^23^, ^43^, ^44^ and p‐tau (Thr231).^22^ In this study, we examined p‐tau (Ser202/Thr205) using immunohistology and measured levels of p‐tau (Thr181) and p‐tau (Thr205) using ELISA. We found the presence of p‐tau (Ser202/Thr205) aggregated into NT and NFT at different levels in the brain biopsies. ELISA immunoassays for p‐tau (Thr181) and p‐tau (Thr205) showed that their abundance closely matched the signal seen in the immunohistology. This evidence agrees with previous studies showing the presence of p‐tau (Ser202/Thr205) associated NT and NFT structures in histological preparations from brain tissues resected from patients with different types of refractory epilepsy,^22^, ^23^, ^24^, ^26^, ^42^, ^43^, ^44^ though a recent study found that only 2 of 56 epilepsy brain resections showed immunostaining for p‐tau (Ser202/Thr205).^27^ In studies by Gourmound et al. (2020) (n = 8) and Tai et al.^26^ (n = 21) high levels of p‐tau significantly correlated with lower cognitive scores.^22^, ^26^ Here we found no significant correlations between p‐tau (Thr205) and p‐tau (Thr181) relative to the FSIQ scores (n = 8). Differences between our findings and others may be due to the different p‐tau residues assessed as well as the approaches used to measure the relative abundance of p‐tau protein which included immunostaining in Tai et al.,^26^ immunoblots in Gourmound et al.,^22^ and ELISA immunoassays in our study. Additional variables may be associated with the brain region studied (hippocampus vs. cortex) and type of cognitive scores utilized for the correlation analysis in the different studies.

We found noticeable Aβ protein in 8 of 12 samples with one biopsy showing vast Aβ plaque accumulation (Figure 4). Other studies reported Aβ immunoreactivity in 2 of 15 epilepsy cases^23^ and 4 of 56 epilepsy patients.^27^ Gourmaud et al.^22^ showed significant increases in amyloid precursor protein and amyloid protein cleavage products in 19 TLE cases^22^ and reported a strong and significant negative correlation between cognitive scores and Aβ protein levels in an analysis of 8 patients with neurocognitive data.^22^ In a similar sample size with neurocognitive data (n = 8), we did not find a significant correlation between the abundance of Aβ protein and the FSIQ scores in the epilepsy cases.

Although we only found a moderate positive correlation coefficient between epilepsy duration and tau hyperphosphorylation, observations from experimental epilepsy support that neuronal hyperexcitability and seizures can induce tau phosphorylation.^45^, ^46^, ^47^, ^48^ In rodent models of neuronal hyperactivity, status epilepticus (SE), and chronic epilepsy, increases in p‐tau and/or Aβ accumulation have been reported.^45^, ^46^, ^47^, ^48^, ^49^ An episode of SE provoked acute and long‐lasting alterations in the levels of tau protein and its phosphorylation.^45^, ^46^, ^47^, ^48^ Time course analyses done by different groups support an initial decrease in tau phosphorylation between 2 and 6 hours post‐SE in whole brain homogenates,^47^ with increases in hippocampal tissue during the epileptogenesis period^45^, ^46^, ^48^, ^49^ and in the chronic epilepsy phase.^45^, ^46^, ^49^ Canet et al.^45^ reported tau hyperphosphorylation in both seizure foci and non‐injured areas in a mouse model of acquired epilepsy, thereby supporting that propagating seizure activity may promote AD‐like pathology in the epileptic brain. This finding further supports our observation that p‐tau and Aβ immunoreactivity localized to areas of abnormal neuronal densities and organization as well as to normal appearing regions of the refractory epilepsy brain biopsies.

Underlying causes of tau hyperphosphorylation in epilepsy could be related to altered activation of intracellular signaling cascades including mTOR.^35^, ^50^ We found moderate to strong positive correlation coefficient values between p‐tau and p‐S6 (Ser235/246) that support a possible relation between these molecules. In fact, seizures as well as mTOR activation have been associated with AD pathology in a 5XFAD mouse model of AD,^35^ in the neuronal specific‐PTEN knockout mouse model of FCD,^50^ and in patients with AD or TLE.^22^, ^23^, ^24^, ^25^, ^26^, ^35^ Other signaling cascades associated with tau and/or amyloid dysregulation include cyclin‐dependent kinase 5 (CDK5),^45^, ^48^ glycogen synthase kinase‐3β pathways (GSK‐3β),^45^, ^48^ protein phosphatase 2A (PP2A) signaling^49^ as well as Wingless/Integrated (Wnt), mitogen‐activated protein kinase (MAPK), and c‐Jun N‐terminal kinase (JNK) signaling, which are known to be altered in epilepsy.^45^, ^48^, ^49^, ^51^, ^52^, ^53^, ^54^ These findings suggest that seizure‐induced disruptions in different signaling cascades, in addition to mTOR, may participate in the generation of abnormal tau and Aβ deposits in epilepsy. Furthermore, an important aspect to consider is the possibility of a sex‐dependent effect on mTOR activation and regulation of tau or Aβ protein levels. In female AD patients and mouse models of AD, the severity of the neuropathology could be attributed to impaired autophagy, a process modulated by mTOR and critical in Aβ plaque clearance.^55^

It must be noted that working with human brain tissues has limitations. In humans, the complexity of neural connections and biochemical profiles can be guided by lifestyle, health conditions, and age, among others. Thus, any of these factors in addition to a history of treatment with antiseizure medication (ASM) could influence the outcome measures of this study. Some ASM have been reported to produce adverse cognitive effects such as a decline in memory, attention, and motor function on people with epilepsy.^56^ ASMs can also contribute to the modulation of mTOR signaling, as well as other intracellular signaling pathways, therefore provoking additional side effects.^57^ In addition, we only examined cortical brain biopsies and neurocognitive scores from 12 cases with refractory epilepsy, which we did not compare to brain samples from non‐epileptic individuals or from AD patients. Another limitation is that tissue homogenates used for biochemical approaches contained undetermined amounts of gray and white matter, which may have different abundances of the proteins studied here. All these factors, along with the possibility of undiagnosed early‐onset AD, can have an impact on the extent of regulation and dysregulation of the p‐tau, Aβ, and/or mTOR pathology found in this group of cases. Thus, additional research is still required to fully understand the potential relationship and impact that p‐tau and/or Aβ aggregation may have in the cognitive dysfunction that can occur in epilepsy.

## CONFLICT OF INTEREST STATEMENT

None of the authors has any conflict of interest to disclose.

## ETHICS STATEMENT

We confirm that we have read the Journal's position on issues involved in ethical publication and affirm that this report is consistent with those guidelines.

## Supporting information


Figure S1
