# Supplementary material for: Assessment of tau phosphorylation and β‐amyloid pathology in human drug‐resistant epilepsy
Source: Epilepsia Open. 2023 Apr 24;8(2):609–22. doi: 10.1002/epi4.12744 (PMC10235185; doi:10.1002/epi4.12744)
Supplement: Supplementary file 1 — Figure S1 [file EPI4-8-609-s001.docx]

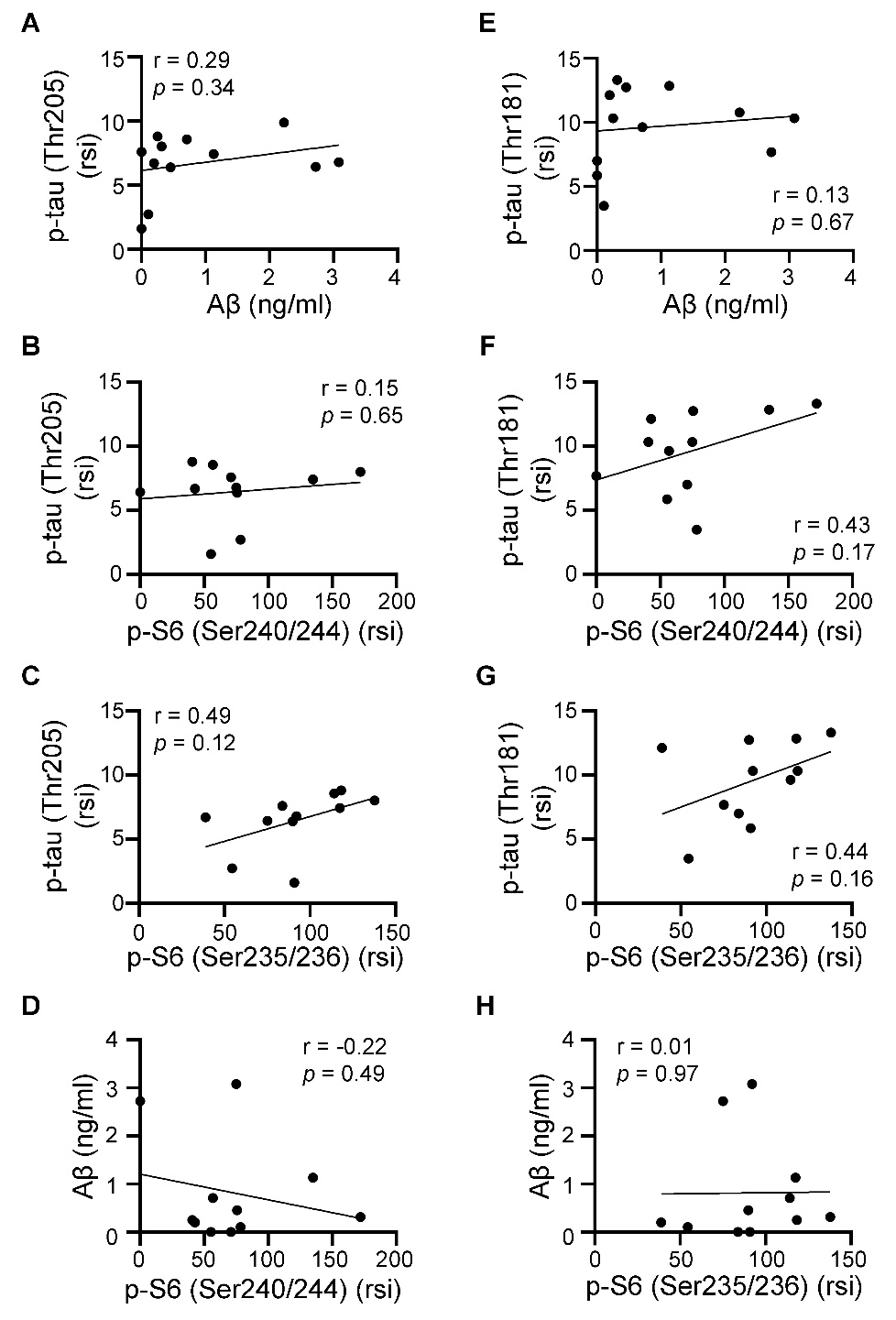


**Supplementary Figure 1. Correlation analysis between p-tau, Aβ, and p-S6 in human refractory epilepsy.** Pearson correlation coefficient (r) analysis between p-tau (Thr205) and Aβ (**A**), p-S6 (Ser240/244) (**B**), and p-S6 (Ser235/236) (**C**). Correlation between Aβ and p-S6 (240/244) (**D)**. Correlations between p-tau (Thr181) and Aβ (**E**), p-S6 (Ser240/244) (**F**), and p-S6 (Ser235/236) (**G**). Correlation between Aβ and p-S6 (235/236) (**H)**.
